# Extracellular vesicles are increased in the serum of children with autism spectrum disorder, contain mitochondrial DNA, and stimulate human microglia to secrete IL-1β

**DOI:** 10.1186/s12974-018-1275-5

**Published:** 2018-08-27

**Authors:** Irene Tsilioni, Theoharis C. Theoharides

**Affiliations:** 1 Department of Immunology, Tufts University School of Medicine, 136 Harrison Avenue, Suite J304, Boston, MA 02111 USA; 2 Sackler School of Graduate Biomedical Sciences, Tufts Medical Center, Tufts University School of Medicine, Boston, MA USA; 3 Department of Internal Medicine, Tufts Medical Center, Tufts University School of Medicine, Boston, MA USA; 4 Department of Psychiatry, Tufts Medical Center, Tufts University School of Medicine, Boston, MA USA

**Keywords:** Autism spectrum disorder, Brain, Exosomes, Extracellular vesicles, Inflammation, IL-1β, Microglia, Mitochondrial DNA

## Abstract

**Background:**

Autism spectrum disorder (ASD) has been associated with brain inflammation as indicated by the activation of microglia, but the triggers are not known. Extracellular vesicles (EVs) are secreted from many cells in the blood and other biological fluids and carry molecules that could influence the function of target cells. EVs have been recently implicated in several diseases, but their presence or function in ASD has not been studied.

**Methods:**

EVs were isolated from the serum of children with ASD (*n* = 20, 16 males and 4 females, 4–12 years old) and unrelated age and sex-matched normotypic controls (*n* = 8, 6 males and 2 females, 4–12 years old) using the exoEasy Qiagen kit. EVs were characterized by determining the CD9 and CD81 membrane-associated markers with Western blot analysis, while their morphology and size were assessed by transmission electron microscopy (TEM). Human microglia SV40 were cultured for 24 h and then stimulated with EVs (1 or 5 μg/mL), quantitated as total EV-associated protein, for 24 or 48 h. IL-1β secretion was measured by ELISA. The results were analyzed using the Mann-Whitney *U* non-parametric test, and all statistical analyses were performed using Graph Pad Prism 5.

**Results:**

EVs were isolated and shown to be spherical structures (about 100 nm) surrounded by a membrane. Total EV-associated protein was found to be significantly increased (*p* = 0.02) in patients as compared to normotypic controls. EVs (5 μg/mL) isolated from the serum of patients with ASD stimulated cultured human microglia to secrete significantly more of the pro-inflammatory cytokine interleukin IL-1β (163.5 ± 13.34 pg/mL) as compared to the control (117.7 ± 3.96 pg/mL, *p* < 0.0001). The amount of mitochondrial DNA (mtDNA7S) contained in EVs from children with ASD was found to be increased (*p* = 0.046) compared to the normotypic controls.

**Conclusions:**

These findings provide novel information that may help explain what triggers inflammation in the brain of children with ASD and could lead to novel effective treatments.

## Introduction

Autism spectrum disorder (ASD) is a pervasive neurodevelopmental disorder characterized by deficits in communication, as well as the presence of restricted, repetitive behaviors [1–3]. The prevalence of ASD in the USA was recently estimated to be 1 in about 59 children [4]. Numerous gene mutations have been identified in patients with ASD, but no direct link has so far been uncovered [5–7]. The lack of reliable biomarkers [8] and specific pathogenesis for ASD [9], as well as the existence of subgroups or comorbidities [10], makes the diagnosis, staging, and treatment of ASD difficult. As a result, the annual economic burden for ASD was estimated at $268 billion for 2015 and projected at $416 billion in 2025 [11].

Abnormal microglial growth and activation indicating inflammation has been reported in the brain of patients with ASD since 2005 [12–16]. However, what stimulates microglia remains unknown. Some of the triggers for microglia activation could be mediators secreted from mast cells (MC), [17, 18] interaction of which with microglia is considered to have an important role in neuroinflammation [19]. One trigger of microglia could be mitochondrial DNA (mtDNA), which we had previously reported to be secreted from stimulated MC [20], and shown to be increased in the serum of children with ASD [20].

Triggers for microglia activation could be carried in the extracellular vesicles (EVs) that are secreted in the intercellular space by diverse cell types [21]. EVs are generated from the cell either when multivesicular bodies (MVBs) fuse with the plasma membrane or they are released directly from the plasma membrane. EVs can be isolated from the serum, plasma, urine, and other biological fluids and can be separated depending on their size (50–1000 nm) with exosomes being on the smaller size range (50–100) [22, 23]. EVs have been shown to contain RNA, DNA, lipids, or proteins [24] that are delivered to the surrounding cells or carried to distal sites [25, 26]. EVs can also be directed to specific cells via targeting proteins in their envelope [27, 28]. Consequently, EVs have the potential to transmit, worsen, or improve disease [29, 30].

Here, we report that serum from children with ASD contains significantly increased EVs as compared to healthy normotypic controls, that these EVs contain mtDNA, and that they can stimulate human-cultured microglia to secrete the pro-inflammatory cytokine interleukin-1β (IL-1β). We chose to study IL-1β because we previously reported that this cytokine is secreted from human-cultured microglia in response to the peptide neurotensin (NT), which we had shown to be increased in the serum of children with ASD [31, 32].

## Methods

### Human subjects

Caucasian children (*n* = 20, 16 males and 4 females, 4-12 years old) diagnosed with ASD were evaluated as part of a clinical trial that was conducted at the Attikon General Hospital, Athens Medical School, Athens, Greece [33]. Children were diagnosed with ASD based on clinical assessment and corroborated by meeting the cutoff scores on both the DSM-IV-TR symptom list and the Autism Diagnostic Observation Schedule (ADOS) algorithm. Subjects were medication-free prior to a blood draw for at least 2 weeks for all psychotropic medications (4 weeks for fluoxetine or depot neuroleptics).

The exclusion criteria were (a) any medical condition likely to be etiological for ASD (e.g., Rett syndrome, fragile X syndrome, or tuberous sclerosis); (b) any neurologic disorder involving pathology above the brain stem, other than uncomplicated non-focal epilepsy; (c) contemporaneous evidence, or unequivocal retrospective evidence, of probable neonatal brain damage; (d) any genetic syndrome involving the CNS, even if the link with autism was uncertain; (e) clinically significant visual or auditory impairment, even after correction; (f) any circumstances that might possibly account for the picture of autism (e.g., severe nutritional or psychological deprivation); (g) mastocytosis (including urticaria pigmentosa); (h) history of upper airway diseases; (i) history of inflammatory diseases; and (j) history of any allergies [33]. This protocol was approved by the Attikon Hospital Human Investigation Review Committee, and all parents or legal guardians provided written informed consent.

### Serum collection

Fasting blood was collected in serum separator vacutainer tubes (BD Biosciences, Rockville, MD). Whole blood was allowed to clot at room temperature for about 15–30 min. The samples were then centrifuged at 1000–2000×*g* for 10 min at 4 °C. The upper clear fraction (serum) was carefully removed and aliquoted (0.5 mL/tube) into clean plastic capped tubes. Serum was also collected from normally developing, healthy Caucasian children (*n* = 20, 16 males and 4 females, 4–12 years old), unrelated to the ASD subjects, who were seen for routine health visits at the Pediatric Department of the Social Security Administration (IKA) polyclinic. All ASD and control blood samples were prepared immediately and stored at − 80 °C. They were later shipped on dry ice to Tufts University for further analysis.

All blood samples were labeled only with a code number, as well as the age and sex of the respective subject. They were sent to Tufts blind without any other identifiers, such as weight, or severity of ASD in the case of patients.

### EV isolation and purification

Total EVs were isolated and purified from 1 mL of serum using the exoEasy Maxi Kit (Qiagen, Valencia, CA) according to the manufacturer’s instructions. Briefly, serum samples were filtered to exclude particles larger than 0.8 μm using syringe filters (EMD Millipore, Burlington, MA). Pre-filtered samples were then mixed with Buffer XBP (EMD) and were bound to an exoEasy membrane affinity spin column. The bound EVs were washed with Buffer XWP (EMD), were eluted with 400 μL Buffer XE (an aqueous buffer containing primarily inorganic salts, EMD), and were then ready for further analysis.

### Transmission electron microscopy

One drop (5 μL) of a sample containing EVs was floated on the grid storage box for 1 min. The grid was then moved to a drop of double-distilled water, and the excess liquid was removed with a filter paper and stained by floating on a small drop of uranyl formate 0.75% for 30 s. After removing the excess uranyl formate with a filter paper, the grids were examined in a TecnaiG^2^ Spirit BioTWIN TEM (FEI Company, Hillsboro, OR, USA), and images were recorded with an AMT 2k CCD camera at a primary magnification of × 20,000–50,000 (Harvard Medical School’s Electron Microscopy Core Facility).

### BCA assay

The concentration of EV total protein was quantified by the bicinchoninic acid (BCA) assay (Thermo Fisher Inc., Rockford, IL) using bovine serum albumin (BSA) as standard.

### Western blot analysis

Extracellular vesicle-associated markers (CD9 and CD81) were determined by Western blot analysis. Protein samples of 10 μg were loaded, separated on 4–12% NuPAGE Bis-Tris gels under SDS-denaturing conditions (Invitrogen Life Technologies, Grand Island, NY) starting by 65 V for 45 min, and then increased to 90 V for another 30 min. Proteins were then electrotransferred onto nitrocellulose membranes (Bio-Rad, Hercules, CA) followed by blocking for 1 h using 5% BSA in Tris-buffered saline containing 0.05% Tween-20. The membranes were then incubated overnight at 4 °C with the following primary antibodies at 1:1000 dilutions: CD9 and CD81 (System Biosciences, Mountain View, CA). For detection, the membranes were incubated with the appropriate secondary horseradish peroxidase (HRP)-conjugated antibody (System Biosciences) at 1:20,000 dilutions for 1 h at room temperature, and the blots were visualized by enhanced Super Signal West Pico Chemiluminescence (Fisher Scientific, Pittsburgh, PA).

### Cell culture

The immortalized human microglia-SV40 cell line derived from primary human microglia was purchased from Applied Biological Materials Inc. (ABM Inc., Richmond, BC, Canada) and cultured in Prigrow III medium (ABM Inc., Richmond, BC, Canada) supplemented with 10% fetal bovine serum (FBS) and 1% penicillin/streptomycin in type-I collagen-coated T25-flasks (ABM Inc.). Microglia-SV40 maintained a specific phenotype and proliferation rate for over ten passages, during which all experiments were performed using multiple microglia thaws and sub-cultured cells. Experiments were carried out in type-I collagen-coated plates (BD PureCoat™ ECM Mimetic Cultureware Collagen I peptide plates (Becton Dickinson, Bedford, MA)). Microglia-SV40 were seeded in 6-well plates (1.0 × 10^5^ cells/well) for 24 h before the stimulation with EVs. Lipopolysaccharide (LPS) or the peptide neurotensin (NT) was used as “positive” controls. Cells were stimulated for 24–48 h, and secreted IL-1β was measured in supernatant fluids using ELISA. Cell viability was determined by Trypan blue (0.4%) exclusion.

### Enzyme-linked immunosorbent assay

IL-1β secretion in supernatant fluids was determined in duplicate by using commercially available ELISA kits (R&D Systems, Minneapolis, MN) according to the manufacturer’s instructions. For all experiments, the control cells were treated with an equal volume of culture medium. The minimum detectable level for IL-1β by ELISA was 5 pg/mL.

### Total DNA isolation and mitochondrial DNA analysis

Total DNA was extracted from EVs using Qiagen DNA Micro extraction kit (Qiagen, CA). Mitochondrial-specific DNA for 7S (mtDNA7S) was detected and quantified by real-time PCR (RT-PCR) using TaqMan gene expression assays (Mt-7S: Hs02596861_s1; GAPDH: Hu, VIC, TAMRA, Applied Biosystems, Carlsbad, CA). Samples were run at 45 cycles using Applied Biosystems 7300 Real-Time PCR System. GAPDH DNA was used to exclude any genomic “contamination.” The same amount of EV-associated DNA (50 ng/μL) from each individual ASD and control sample was used for the analysis of mtDNA.

### Statistical analysis

The concentration of EV-associated proteins was compared between the control and ASD samples using the Mann-Whitney *U* non-parametric test following the examination of normality of distribution using Shapiro-Wilk’s test.

All in vitro conditions were performed in triplicate, and all experiments were repeated at least three times (*n* = 3). Results are presented as mean ± SD. Data from stimulated and control samples were compared using the unpaired two-tailed Student’s *t* test. The significance of comparisons is denoted by *p* < 0.05. All analyses were performed using Graph Pad Prism 5.

## Results

### EV characterization

**Fig. 1:**
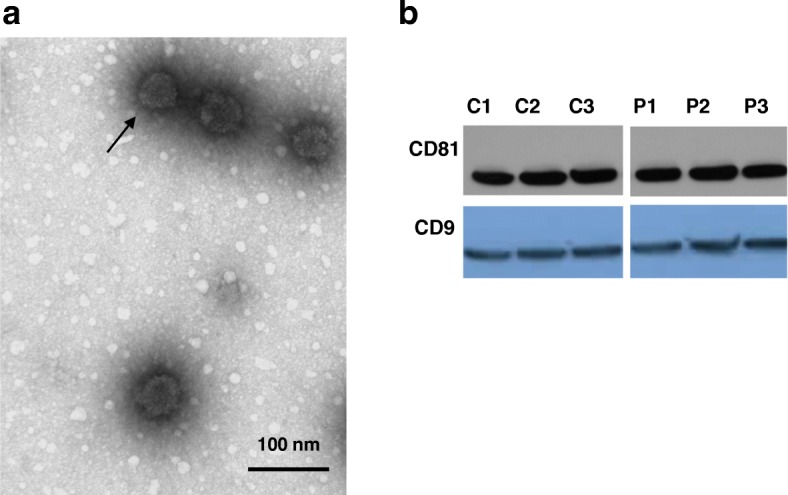
Characterization of EVs. **a** The morphology of EVs from the serum of patients with ASD was evaluated using TEM (scale bar, 100 nm); the arrow points to the EV membrane. **b** EVs collected from the serum samples of normotypic controls (*n* = 3) and ASD children (*n* = 3) were characterized by Western blot analysis for the presence of the EV-associated proteins, CD81 and CD9. C = controls, *P* = patients

The morphology of EVs was evaluated using TEM. EVs had a spherical shape with a size of approximately 100 nm surrounded by a membrane (Fig. 1a). The identity of EVs was further validated by quantitating the EV membrane-associated markers CD9 and CD81 using Western blot analysis (Fig. 1b). There was no apparent difference in the size or shape of EVs between the control and ASD samples.

### Serum EV-associated protein is increased in children with ASD

**Fig. 2:**
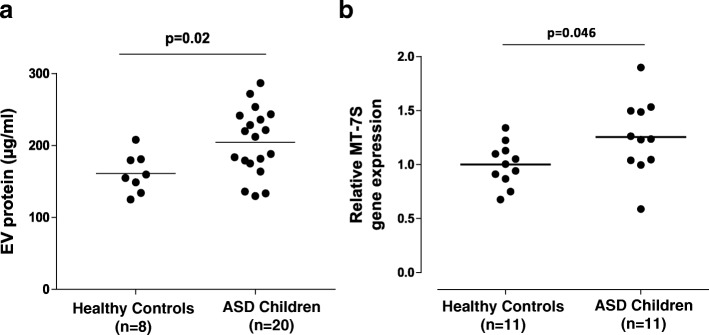
Scattergrams presenting EV-associated protein and mtDNA in the serum of children with ASD compared to normotypic controls. **a** Total EV-associated protein isolated from the serum samples of children with ASD (*n* = 20) compared to healthy normotypic controls (*n* = 8). **b** Serum EV-associated mtDNA7S gene expression values. Levels of mtDNA7S were assayed by qPCR and normalized to the mean of all control samples. GAPDH was undetectable

The total protein concentration in EV-enriched serum samples was significantly higher in children with ASD (204.6 ± 10.6 μg/mL) as compared to the normotypic controls (161.4 ± 9.6 μg/mL), *p* = 0.02 (Fig. 2a).

### Serum EV-associated mitochondrial DNA

We also measured the EV-associated mtDNA7S in ASD children and normotypic controls. The amount of mtDNA7S contained in EVs from children with ASD was found to be increased (*p* = 0.046) compared to the normotypic controls (Fig. 2b).

### EVs stimulate secretion of IL-1β from human microglia

**Fig. 3:**
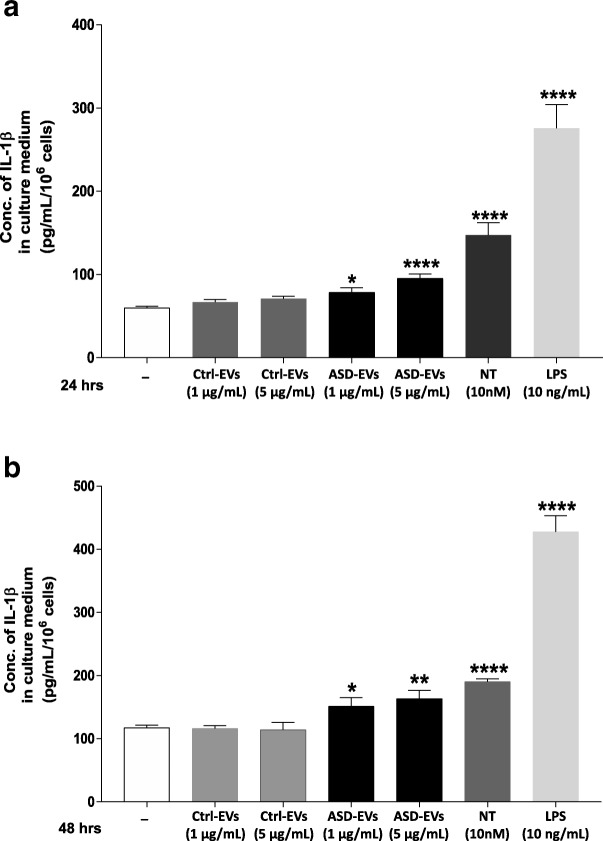
Serum-derived EVs stimulate IL-1β secretion from human microglia. Immortalized HM-SV40 microglia (1.0 × 10^5^ cells) were stimulated with serum-derived EVs, quantitated as total EV-associated protein (1 or 5 μg/mL), from patients with ASD for **a** 24 h and **b** 48 h. Secretion of IL-1β was measured in the supernatant fluid by specific enzyme-linked immunosorbent assay (ELISA). All conditions were performed in triplicates for each data set and repeated three times (*n* = 3). Microglia were also stimulated with NT (10 nM) or LPS (10 ng/mL) used as “positive” controls. The significance of comparisons of each experimental condition to the control unstimulated cells is denoted by *p < 0.05, **p < 0.001, or ***p < 0.0001. Conc = concentration; ctrl = control

We then evaluated the effect of EVs derived from the serum of patients with ASD on human-cultured microglia secretion of IL-1β. We chose to measure this pro-inflammatory cytokine because we recently reported that microglia could secrete IL-1β in response to NT, which we had shown to be increased in the serum of children with ASD [31, 32]. Human-immortalized microglia SV40 were stimulated with EVs (1 or 5 μg/mL), quantitated as total EV-associated protein, for 24 or 48 h, and IL-1β secretion was measured by ELISA. EVs from each ASD and control sample were used separately and were not pooled. Both 1 and 5 μg/mL stimulated significant secretions of IL-1β (Fig. 3). Stimulation with 5 μg/mL for 24 h and 48 h significantly increased the secretion of IL-1β to 95.36 ± 5.31 pg/mL and 163.5 ± 13.34 pg/mL, respectively, as compared to control microglia (59.76 ± 2.03 pg/mL, *p* < 0.0001, and 117.7 ± 3.96 pg/mL, *p* = 0.008, respectively) (Fig. 3a–b). Lower amount of EVs (1 μg/mL) also stimulated IL-1β secretion at 24 h (78.47 ± 5.46 pg/mL, *p* = 0.02) and at 48 h (151.3 ± 13.63 pg/mL, *p* < 0.04), respectively (Fig. 3a–b).

EVs from normotypic children did not have a significant effect (*p* = 0.09) at either 1 or 5 μg/mL, as compared to the unstimulated control cells. Neurotensin (10 nM) and LPS (10 ng/mL) used as “positive” controls also increased (*p* < 0.0001) the secretion of IL-1β (Fig. 3).

## Discussion

This is the first report, to the best of our knowledge, that serum from children with ASD contains significantly increased total EV-associated protein as compared to the healthy normotypic controls. The size of the EVs qualifies them as exosomes. There was no apparent difference in shape, size, or the EV-associated proteins CD9 and CD81 between ASD and normotypic controls.

We also show that serum-derived EVs from children with ASD stimulate the secretion of the pro-inflammatory cytokine IL-1β from human SV40-immortalized microglia. We chose to measure IL-1β because we recently reported that the neuropeptide neurotensin (NT) stimulates human microglia to secrete IL-1β, [18] which has been shown to be increased in the brains of children with ASD, [34] and in a mouse model of autism [35].

EVs from children with ASD contain a significant amount of mtDNA compared to the normotypic controls. We had previously reported that mtDNA is increased in children with ASD [20]. We also reported that mtDNA can be secreted extracellularly from mast cell stimulated by the neuropeptide substance P (SP) [20], an important finding given that atopic diseases have been associated with increased risk of ASD [36]. Extracellular mtDNA serves as an alarmin and leads to pro-inflammatory mediator secretion from immune cells [37, 38]. In the case of ASD, mtDNA may serve as an “innate” pathogen [39] that would be protected from degradation inside EVs and could reach the microglia through the brain lymphatics [40] or through the blood-brain barrier [41].

IL-1β synthesis occurs via activation of the Nod-like receptor pyrin domain-containing protein 3 (NLRP3) inflammasome which requires two different signals [42]. In our findings, signal 1 could be mtDNA, but EVs must contain some other molecule that could serve as signal 2. A possible candidate could be the peptide NT. Our laboratory previously reported NT to be increased in the serum of children with ASD [31, 32] and which we recently reported can stimulate secretion of IL-1β from human microglia [18].

The cellular origin of the increased serum EVs is presently unknown. Astrocytes and glioblastoma cells have been reported to release EVs containing mtDNA [24], but there is no evidence at present that the serum EVs derive from the brain. Serum EVs could derive from MC [43] since MC-microglia interactions are considered important in the inflammation of the brain [19, 44].

Microglia-derived “microparticles,” a specific type of extracellular vesicles, are also released after a traumatic brain injury (TBI) and can activate microglia in vitro [45]. LPS can activate microglia to release “microparticles” with increased content of pro-inflammatory mediators IL-1β and miRNA-155 in vitro [45]. EVs could also potentially transport environmental triggers [46] or derive from other comorbid conditions, [10, 47] but such were not present in the patients with ASD analyzed.

In addition to mtDNA, mRNA and miRNA can be transported by EVs and have been associated with brain inflammation and multiple sclerosis [48]. In fact, a number of miRNAs such as miR-27a, miR-23a, and miR-628-5p were detected in the saliva of children with ASD [49]. One study reported differential expression of miR-497 in EVs isolated from postmortem pre-cortex from patients with schizophrenia and bipolar disorder [50]. EVs were also shown to contain several proteins or miRNA [51] that may be involved in Alzheimer’s and in Parkinson’s disease with dementia [52, 53]. EVs are increasingly discussed in the context of neurodegenerative diseases [22, 54].

## Conclusion

To the best of our knowledge, this is the first report describing that serum from children with ASD contains significantly increased total EV-associated protein as compared to healthy normotypic controls, that these EVs contain mtDNA, and that they can stimulate human-cultured microglia to secrete the pro-inflammatory cytokine IL-1β. Our results, although preliminary, provide novel information that may help explain the inflammation of the brain and ASD pathogenesis. Further studies on a larger sample size are needed to extend these findings and validate their usefulness for diagnosis and use as a target for novel effective treatments for ASD.

## Abbreviations

ADOS: Autism Diagnostic Observation Schedule
ASD: Autism spectrum disorder
EVs: Extracellular vesicles
IL-1β: Interleukin-1β
MC: Mast cells
mtDNA: Mitochondrial DNA
MVBs: Multivesicular bodies
NLRP3: Nod-like receptor pyrin domain-containing protein 3
NT: Neurotensin
SP: Substance P
TEM: Transmission electron microscopy
